# A Spatially Propagating Biochemical Reaction**

**DOI:** 10.1002/anie.201005638

**Published:** 2010-12-22

**Authors:** Xiaoli Liao, Rafe T Petty, Milan Mrksich

**Affiliations:** Department of Chemistry, University of Chicago929 East 57th Street, Chicago, IL 60637 (USA)

**Keywords:** adaptor domains, autocatalysis, monolayers, phosphorylation, propagation

Unlike reactions performed in the laboratory—which use homogeneous solutions having spatially uniform concentration profiles of each reactant—reactions that occur in biological systems often involve reactants that are present in concentration gradients and can display unexpected kinetic properties.^1^–^5^ Such biological reactions can generate spatio-temporal structures that derive from the inclusion of feedback, autocatalysis, and other non-linear influences on rate.^6^–^9^ While these examples are prevalent in biology, it is still challenging to engineer such systems in the laboratory.^10^–^13^ Here, we demonstrate a reaction wherein a soluble kinase enzyme phosphorylates a peptide that is immobilized to a self-assembled monolayer (SAM) and that proceeds with a spatially organized propagation of the product. This spatial control over the reaction derives from an autocatalytic feature that operates at the boundary between the substrate and product.

Our system is based on Abelson tyrosine kinase (Abl) which has both a catalytic domain and a Src homology 2 (SH2) domain that binds to the phosphopeptide product of the phosphorylation reaction. For example, Abl phosphorylates the substrate peptide AIYENPFARKC to give AIpYENPFARKC (which we abbreviate as Y and pY, respectively), and the kinase can then bind to pY. Previously, we used SAMs of alkanetiolates on gold to demonstrate that Abl can operate autocatalytically on an immobilized substrate (Figure 1 a).^14^ The SAMs presented a peptide substrate for Abl kinase against a background of tri(ethylene glycol) groups, which render the monolayers inert to protein adsorption,^15^ and the peptides could be characterized with matrix-assisted laser desorption–ionization mass spectrometry (i.e., the SAMDI method).^16^–^18^ The autocatalytic kinetic property arises due to the binding of the phosphopeptide product to the SH2 domain of the kinase, which then recruits the kinase to the surface, giving an approximately 30-fold increase in the rate for phosphorylation of nearby peptides.^14^ Hence, the phosphorylation reaction is fastest in those regions of the monolayer that are directly adjacent to the regions that present product. Here, we explicitly characterize the spatial propagation of the reaction product and we also show that patterned monolayers display initial rates that depend on the geometrical features of the pattern, but not the relative amounts of substrate and product.

**Figure 1:**
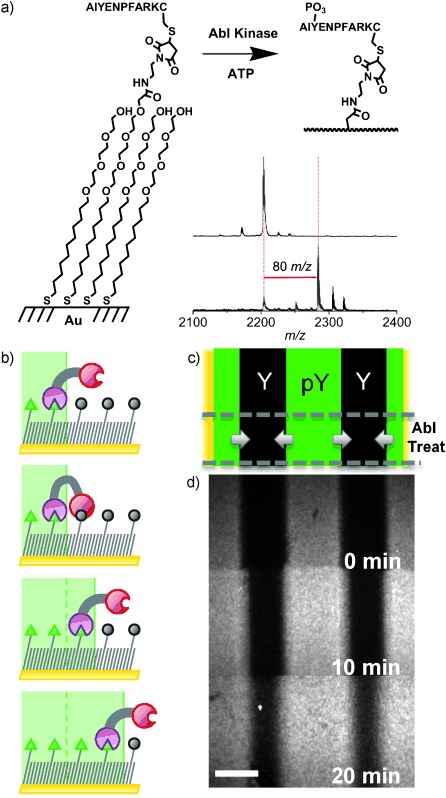
a) Treatment of a monolayer that presents a peptide with Abl kinase and ATP results in phosphorylation of the peptide, which can be detected by mass spectrometry that reveals the corresponding mass change of 80 *m*/*z*. b) Schematic of the reaction front propagating away from regions initially patterned with the phosphopeptide, where the reaction front is indicated in green, the SH2 domain of Abl kinase in purple, and the catalytic domain in red. c) Regions of phosphopeptide (green) were initially patterned on the surface using an agarose stamp. The remainder of the surface was backfilled with the unphosphorylated peptide (black). d) The patterned surface was treated with Abl kinase for different times, and the reaction propagation was visualized using an antiphosphotyrosine antibody. Scale bar=25 μm.

We patterned a monolayer into alternate regions that presented the phosphopeptide and the peptide substrate. We reasoned that those peptide substrates located immediately adjacent to regions that present phosphopeptide would be phosphorylated most rapidly, because only at the boundary can tethered enzyme interact with the substrate. Hence, we expected that the reaction would show a spatial propagation in time (Figure 1 b). To test this hypothesis and to characterize the spatial velocity with which the reaction proceeds, we used an agarose stamp that was inked with the phosphopeptide to pattern a maleimide-terminated monolayer with a parallel set of striped regions that presented the peptide. We removed the stamp and treated the monolayer with the unphosphorylated peptide to backfill the remaining regions. We then treated the entire surface with a solution containing Abl kinase and ATP. Parallel reactions were performed and individual reactions were stopped at different times and treated with an antibody that binds phosphotyrosine and a fluorescently labeled secondary antibody (Figure 1 c). In this way, we could spatially resolve the phosphorylated peptide on the surface and characterize the time course of the reaction. The bright regions correspond to the phosphorylated peptide and black regions correspond to the unphosphorylated peptide. As expected, the stripe having product broadens with increasing reaction times, and is consistent with a propagation of the reaction from the initially patterned boundary between phosphorylated and unphosphorylated peptide (Figure 1 d).

The reaction propagated over distances greater than 10 µm. We measured a velocity of 0.87 μm min^−1^ for a surface presenting the peptide substrate at a density of 1 % relative to total alkanethiolates. The experiment was repeated using a monolayer that presented the peptide at a density of 0.5 % and we measured a propagation velocity for the reaction of 0.51 μm min^−1^ (Figure 2 a). This lower velocity is consistent with our previous observation that the autocatalytic reaction has a strong dependence on substrate density, and shows a greater rate increase for monolayers that present the substrate at higher density.^14^ We have not determined whether sequential phosphorylation reactions are catalyzed by a single kinase—where the enzyme repeatedly dissociates and reassociates to the newly formed phosphopeptide—or whether new kinase enzymes are recruited from the bulk region. Therefore, we do not know whether the use of an SH2 domain having a higher dissociation rate constant will lead to a larger propagation velocity. In contrast to molecular motors, which move directionally along a pattern of ligand, in our system, the propagating species is not the enzyme itself, but the product peptides, and the propagation direction is determined by the spatial organization of the product and the substrate on the surface.^19^, ^20^ We also tested whether the rate of spatial propagation depends on the intrinsic activity of the kinase enzyme. We used a constant concentration of ATP (200 μm) in the reaction buffer but included increasing concentrations of the non-hydrolyzable ATP analogue adenosine 5′-(β,γ-imido)triphosphate (AMP-PNP) which is a competitive inhibitor for ATP and therefore reduces the turnover number of the kinase. Indeed, we found that the propagation rate decreases with increasing concentrations of AMP-PNP (Figure 2 b), demonstrating that the kinase activity was partially inhibited.

**Figure 2:**
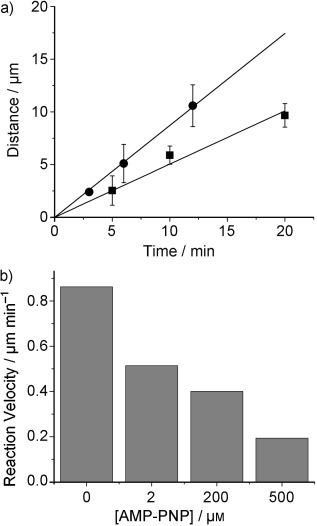
a) The reaction velocity depends on the density of the immobilized peptide substrate. Reactions were stopped after different times and imaged with fluorescence microscopy. A plot of the distance of reaction propagation versus time was linear and has a slope that corresponds to the reaction velocity. ▪: 0.5 % substrate density, d*x*/d*t*=0.51 µm min^−1^, *R*^2^=0.965; •: 1.0 % substrate density, d*x*/d*t*=0.87 µm min^−1^, *R*^2^=0.997. b) The reaction velocity decreases in the presence of the non-hydrolyzable ATP analogue AMP-PNP.

The finding that the reaction proceeds most rapidly at the boundary between phosphorylated and unphosphorylated peptides suggests that relative rates for a reaction on patterned surfaces would depend on the spatial arrangement of the pattern and not only on the ratio of the two peptides on the surface. If the rate is averaged over a region of the surface that is large compared to the feature sizes of the patterns, we reasoned that it would increase with the “boundary density” of the pattern, defined as the integrated length of the perimeter of phosphopeptide-terminated regions of the monolayer per unit area of the monolayer. To explore this possibility, we utilized four patterns that had the phosphopeptide organized in circular features or zig-zag lines, each at two different sizes (Figure 3 a). The small circles had a larger boundary density than did the large circles, and likewise for the zig-zag lines. We treated the patterned monolayers with kinase for times ranging from 0 to 30 min and then used SAMDI mass spectrometry to analyze the monolayers to determine the average yield of the reaction. We found that the initial rate of reaction (*r*_initial_, again which represent a rate averaged over the patterned region) increased linearly with the boundary density (Figure 3 b). We also investigated how the binding affinity of the phosphopeptide for the SH2 domain affects the rate. We compared the reaction on two patterned monolayers that each presented the same peptide substrate but with one of two different phosphopeptides that differed in their affinity for the SH2 domain. The peptide sequence AIpYENPFARKC (black curve) has a higher affinity for the SH2 domain than does the pYAAPFKC (red curve) (Figure 3 b). We found that the higher affinity phosphopeptide led to a greater initial rate. These results demonstrate that the rate of the reaction can be manipulated by controlling the spatial organization of product and substrate on the monolayer and by adjusting the affinity of the phosphopeptide for the SH2 domain.

**Figure 3:**
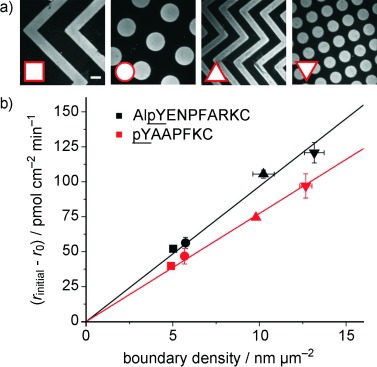
a) Monolayers were patterned with four distinct geometries that had a similar overall ratio of substrate to product, but with varying boundary densities as described in the text. The icon in the lower left of each image corresponds to its position on the graph. b) The reaction kinetics are sensitive to the initial geometry of patterned phosphopeptide, where the initial rate for patterned surfaces (*r*_initial_) increases with the boundary density (*r*_0_ is the initial rate of non-patterned surface, serving as the background rate). The black curve represents the high affinity phosphopeptide and the red curve represents the lower affinity phosphopeptide. Scale bar=100 μm.

This study demonstrates a reaction system that shows a spatial propagation of product, despite the uniform concentration of enzyme catalyst throughout the system. This example is significant because it describes an approach to engineering reactions to give spatio-temporal control over the products and progression of reactions in a system. The use of self-assembled monolayers was important to this work for several reasons.^21^ First, they allow the placement of reactive groups in a uniform environment on the surface and offer good control over the densities and geometric patterns of the reactants. The compatibility of monolayers with a variety of analytical techniques, including mass spectrometry and fluorescence microscopy, allows reaction kinetics and spatial structure to be analyzed, and the use of immobilized reactants is important for eliminating diffusion and effectively stabilizing the spatial distributions of the products. We believe that this and related approaches offer a new opportunity to create reaction systems that share the emergent properties that are so common in biological reaction networks.^22^–^26^

## Notes

Detailed facts of importance to specialist readers are published as ”Supporting Information”. Such documents are peer-reviewed, but not copy-edited or typeset. They are made available as submitted by the authors.
