# Supplementary material for: A Spatially Propagating Biochemical Reaction
Source: Angew Chem Int Ed Engl. 2010 Dec 22;50(3):706–8. doi: 10.1002/anie.201005638 (PMC3154084; doi:10.1002/anie.201005638)
Supplement: Supplementary file 1 [file anie0050-0706-SD1.pdf]

Angewandte Chemie
Eine Zeitschrift der Gesellschaft Deutscher Chemiker

Supporting Information
© Wiley-VCH 2011
69451 Weinheim, Germany

A Spatially Propagating Biochemical Reaction**

*Xiaoli Liao, Rafe T. Petty, and Milan Mrksich**

anie_201005638_sm_miscellaneous_information.pdf

SUPPORTING INFORMATION

Materials and Methods

Synthesis of peptides: Peptides were synthesized at 0.1mmol scale each on Fmoc-Rink amide 4-methylbenzhydrylamine resin (AnaSpec. Inc., San Jose, CA). All peptides were purified by reverse phase HPLC on a C18 column (Waters). All amino acids were purchased from Anaspec (San Jose, CA). Benzotriazole-1-yl-oxy-tris-pyrrolidino-phosphonium hexafluorophosphate (PyBOP) was purchased from Chempep Inc. (Wellington, FL). Piperidine, acetic anhydride, triisopropylsilane (TIS), N,N-dimethylformamide (DMF), and dichloromethane (DCM) were purchased from Sigma-Aldrich (St. Louis, MO). Trifluoroacetic acid (TFA) was purchased from VWR.

Preparation of SAMs Presenting Maleimide Groups: The maleimide-presenting SAMs were prepared as previously reported.[1] Briefly, gold-coated coverslips (4 nm Ti, 22 nm Au) were immersed in an ethanolic solution containing a symmetric disulfide presenting tri(ethylene glycol) groups and an asymmetric disulfide presenting one maleimide group and one tri(ethylene glycol) group at different ratio (in case of 10% maleimide density, a molar ratio of 4:1 was used) overnight with a total concentration of disulfide of 0.2mM.

Kinase assay: Abl kinase (New England Biolabs, Ipswich, MA) was diluted to 1~2 U μL^{-1} in kinase buffer (50 mM Tris-HCl, 10 mM $MgCl_2$, 1 mM EGTA, 2 mM DTT, 0.01% Brij 35, pH 7.5) with ATP (0.2~1 mM), and 1-2 μL was applied to each monolayer (~4 mm^2) and incubated at 30 °C for different time periods.

Mass Spectrometry: Monolayers were treated with matrix (2,4,6-trihydroxyacetophenone, 20 mg/mL in acetonitrile), air dried, and analyzed by SAMDI-MS to produce a mass spectrum for each circle. Mass analysis was performed using a 4800 MALDI-TOF/TOF (Applied Biosystems, Framingham, MA). A 355 nm Nd:YAG laser was used as a desorption/ionization

source, and all spectra were acquired with 20 kV accelerating voltage using positive reflector mode. The extraction delay was 450 ns, 3000 laser shots were applied, and the entire surface of the circle was sampled.

Fabrication of PDMS device: Poly(dimethylsiloxane) (PDMS) devices with microchannels were made from 184 Silicone Elastomer Kit (Dow Corning Sylgard Brand, 10:1 base:curing agent ratio). Holes were punched to make inlets and outlets of the channels using a sharpened 19 Ga hollow needle. Before in-channel peptide immobilization or kinase treatment, the interior surfaces of all PDMS channels were blocked with PBS (pH =7.4) containing 10 mg mL^{-1} bovine serum albumin (BSA) for 30 minutes to 1 hour. The channels were emptied and rinsed with PBS prior to use.

Fabrication of agarose stamp: Preparation of agarose stamp was modified from previously described methods.[2, 3] Briefly, 5% low-melting temperature agarose (w/v%) (gel strength > 1200g cm^{-2}, Fisher Scientific, Pittsburgh, PA) in PBS was heated in a microwave oven and cast onto the PDMS master presenting an array of micro-wells. After gelation at room temperature for more than two hours, the agarose layer was gently peeled off and cut into approximately 8 mm × 8 mm × 6 mm blocks patterned with the microarray of posts, negative features of the array of features in the PDMS master. The stamps were used directly or storage in a bath of deionized water with the posts facing upwards.

Microcontact printing with agarose stamp: The agarose stamps with different features were placed upside down with posts faced upwards) and covered with phosphorylated peptide (0.5mM in PBS, ~10 µL/ stamp). The stamp was briefly dried under N_2 flow after absorbing most of the solution (~5 min) and placed gently onto a maleimide SAM surface. The stamp was removed from the surface after 3-5 minutes and the surface with phosphorylated peptide patterns was

washed with water and ethanol and dried under N_2 flow. The unphosphorylated peptide was applied to the whole surface to backfill the remaining area. The features generated by this method had larger sizes compared to that of the designed features, which led to not exactly the same amount of phosphopeptide relative to the total peptide (%pY in Table S3).

Preparation of patterns by laminar flow: A PDMS stamp with Y-shaped microchannels was sealed to the monolayers and clamped between 2 pieces of ¼" thick polycarbonate using binder clips. Aqueous peptide solutions were introduced to the channels using syringes (Hamilton Co., Reno, NV) attached to the channel by 27 gauge Teflon tubing. Syringe pumps (Harvard Apparatus, Holliston, MA) were used to regulate the fluid flows. The pattern of unphosphorylated and phosphorylated peptide (0.2mM in PBS) was generated in the PDMS channel using fluid flow rate of 40 μL min^{-1} for both peptide solutions. The device was disassembled, rinsed with water, dried under N_2 flow.

In-channel kinase treatment: A PDMS stamp of parallel 6 or 8 channels was then reapplied on top of the SAMs in a perpendicular direction so that each channel intersected the line of peptides immobilized previously. Kinase solution was introduced into the channels for reaction. The channels were emptied and washed with PBS at different time points. The SAMs were disassembled from PDMS after reaction, rinsed with water and dried prior to immunostaining.

Immunostaining: Rabbit anti-phosphotyrosine primary antibodies (Sigma, St. Louis, MO), secondary AlexaFluor 488 anti-rabbit (Molecular Probes, Eugene, OR) were prepared in buffer containing 0.1% BSA (Sigma-Aldrich, St. Louis, MO) with a dilution of 1:250 and 1:400, respectively. Primary staining and secondary antibody staining was done at 30 ºC for 1 hour and 30 minutes, respectively. Slides were mounted in Aqua Poly/Mount (Polysciences, Inc.,

Warrington, PA) with microscope cover glass (thickness 1.5, Fisher Scientific, Pittsburgh, PA) on top.

Imaging and analysis: Slides were images with a Hamamatsu back-thinned EM-CCD camera on an Olympus IX81 spinning disc confocal microscope (Center Valley, PA) using a 40X water-immersion objective. Images were taken with Slidebook (Intelligent Imaging Innovations, Inc., Denver, CO) using identical exposure times to allow accurate comparison of fluorescence intensities between images. Images were exported for analysis in ImageJ (NIH, Bethesda, MD). To measure the distance of propagation, the plot profile of each kinase−treated area was taken to determine the position at the half-maximum fluorescence intensity (the middle point). By subtracting the middle point position of the untreated area, the distance that the kinase reaction propagated was obtained (Figure S1b).

SAMDI data analysis and initial rate comparison: The extent of phosphorylation was was calculated by measuring the relative intensities of each molecular ion peak (M^+): $Y = I_p/(I_p+I_s)$, where p refers to the phosphorylated peak and s refers to the parent peak. For patterned surfaces, the phosphopeptides for patterning and the phosphorylated form of the substrate peptide have different molecule weights. In this way the product generated by kinase reaction is distinguishable from the initially patterned phosphopeptides in SAMDI experiment. The initial rate ($r_{initial}$) for each surface was calculated from the slope of the linear part of the time-course reaction profile. The initial rate of a non-patterned surface (r_0) was included for each experiment as background reactivity.

Supporting Figures and Tables:

a)

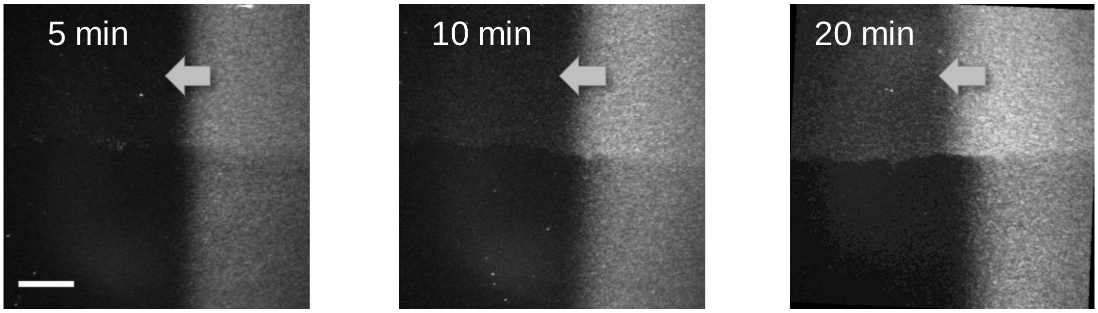

b)

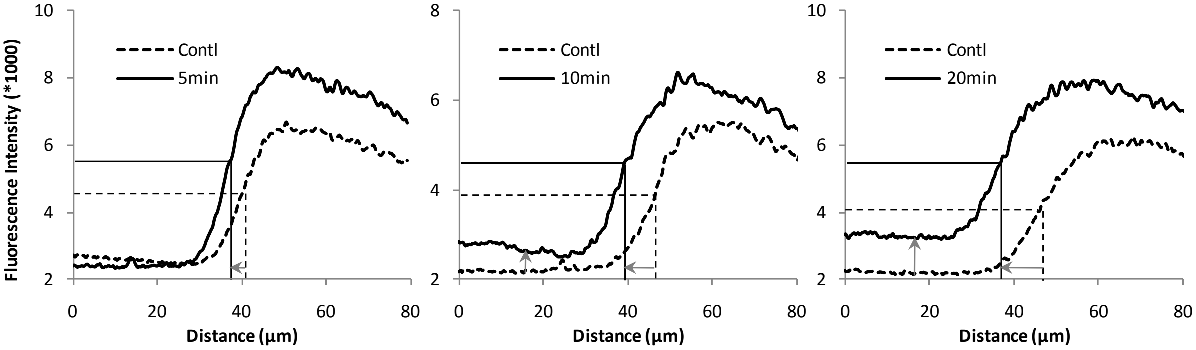

c)

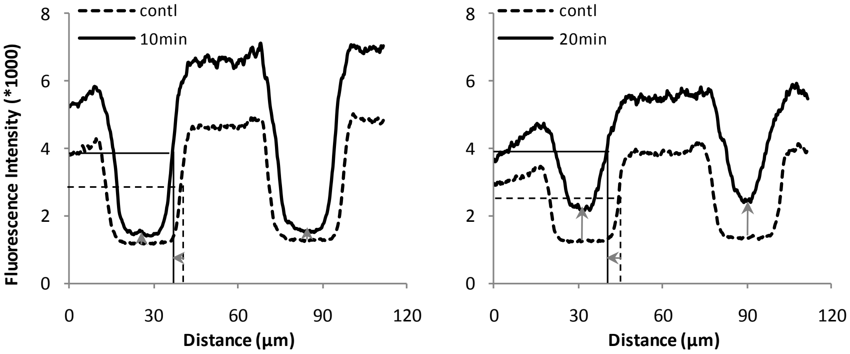

Figure S1. a) Images of laminar flow patterned surface treated with Abl kinase for different times (the upper-panel of each image). The reaction propagation is visualized using an antiphosphotyrosine antibody. Arrows indicate propagation directions (Scale bar = 25 μm). b) The plot profiles of each patterned surface. The positions at the half-maximum fluorescence intensity are used to determine the propagation distances, which are indicated by the horizontal arrows. The vertical arrows indicate the level of the background phosphorylation. c) The plot profiles of agarose microcontact printed surface treated with Abl kinase for different times (fluorescent images in Figure 1d). The horizontal and vertical arrows indicate propagation distance and the level of the background phosphorylation, respectively.

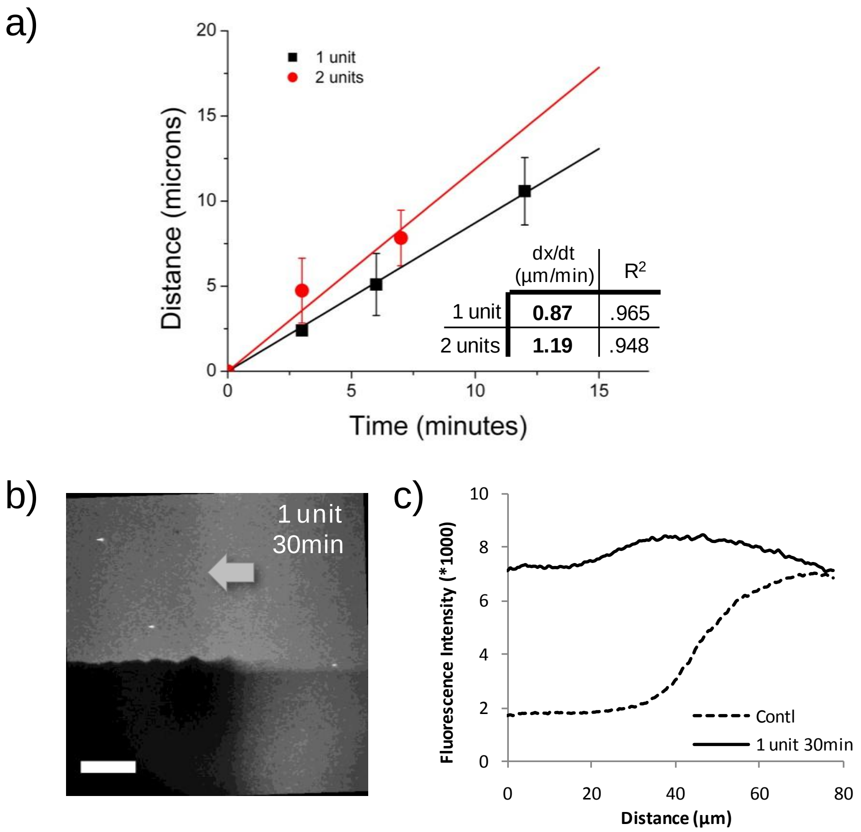

Figure S2. a) The reaction velocity depends on the concentration of the kinase (1 U µL^{-1} vs 2 U µL^{-1}) at the same peptide density (1%). Data are plotted as distance versus time, and the slope corresponds to the reaction velocity. Higher kinase concentration results in faster propagation. b) Images of laminar flow patterned surface treated with 1 U µL^{-1} Abl kinase for 30 minutes at 1% substrate density (Scale bar = 25 µm). Surface treated with 2 U µL^{-1} Abl kinase for 15 minutes showed similar image where the background phosphorylation increases and becomes indistinguishable from the propagation front. c) The plot profile of Figure 1b.

Table S3. The designed feature sizes and the measured average feature sizes (calculated from Image J) of four patterns. Because the patterned phosphopeptide was not included in the reaction and initial rate calculation, the difference in its relative amount would not affect correlation between the initial rate and the boundary density.

Designed feature Size (diameter or width)	Off-set (µm)	Measured average feature Size (µm)	Average pY area	Average Boundary Density (nm µm^{-1})
Line 80 µm	408	89	22%	4.9
Circle 160 µm	320	186	26%	5.7
Line 40 µm	204	53	26%	9.8
Circle 80 µm	160	104	33%	12.8

[1] B. T. Houseman, E. S. Gawalt, M. Mrksich, *Langmuir* **2003**, *19*, 1522.
[2] M. Mayer, J. Yang, I. Gitlin, D. H. Gracias, G. M. Whitesides, *Proteomics* **2004**, *4*, 2366.
[3] B. A. Grzybowski, K. J. M. Bishop, *Small* **2009**, *5*, 22.
